# Comprehensive and semi-quantitative analysis of carboxyl-containing metabolites related to gut microbiota on chronic kidney disease using 2-picolylamine isotopic labeling LC-MS/MS

**DOI:** 10.1038/s41598-019-55600-1

**Published:** 2019-12-13

**Authors:** Yoshitomi Kanemitsu, Eikan Mishima, Masamitsu Maekawa, Yotaro Matsumoto, Daisuke Saigusa, Hiroaki Yamaguchi, Jiro Ogura, Hiroki Tsukamoto, Yoshihisa Tomioka, Takaaki Abe, Nariyasu Mano

**Affiliations:** 1 Department of Pharmaceutical Sciences, Tohoku University Hospital, Sendai, Japan; 2 Department of Clinical Biology and Hormonal Regulation and Division of Nephrology, Endocrinology, and Vascular Medicine, Graduate School of Medicine, Tohoku University, Sendai, Japan; 3 Laboratory of Oncology, Pharmacy Practice and Sciences, Graduate School of Pharmaceutical Sciences, Tohoku University, Sendai, Japan; 4 Department of Integrative Genomics, Tohoku Medical Megabank Organization, Tohoku University, Sendai, Japan; 5 Department of Medical Science, Graduate School of Biomedical Engineering, Tohoku University, Sendai, Japan

**Keywords:** Metabolomics, Kidney

## Abstract

Carboxyl-containing metabolites, such as bile acids and fatty acids, have many important functions and microbiota is involved in the production of them. In the previous study, we found that the chronic kidney disease (CKD) model mice raised under germ-free conditions provided more severe renal damage than the mice with commensal microbiota. However, the precise influence by the microbiome and carboxyl-containing metabolites to the renal functions is unknown. In this study, we aimed to develop a novel chemical isotope labeling-LC-MS/MS method using the 2-picolylamine and its isotopologue and applied the analysis of effects of microbiome and CKD pathophysiology. The developed semi-quantitative method provided the high accuracy not inferior to the absolute quantification. By comparing of four groups of mice, we found that both microbiota and renal function can alter the composition and level of these metabolites in both plasma and intestine. In particular, the intestinal level of indole-3-acetic acid, short-chain fatty acids and n-3 type of polyunsaturated fatty acid, which play important roles in the endothelial barrier function, were significantly lower in germ-free conditions mice with renal failure. Accordingly, it is suggested these metabolites might have a renoprotective effect on CKD by suppressing epithelial barrier disruption.

## Introduction

The mammalian gastrointestinal tract contains trillions of microorganisms, referred to collectively as gut microbiota^1^. The gut microbiota coevolves with the host for a mutually beneficial coexistence by sharing numerous metabolites, including that cannot produce each other. The gut microbiota not only produces certain bile acids as well as short-chain fatty acids (SCFA) and indole-3-acetic acid (IAA) but also affect a fatty acid metabolism of the host^2–5^. Bile acids, IAA and fatty acids are groups of carboxylic acids and play important roles in many metabolic pathways^6–8^. The production of these metabolites by microbiomes contributes to the host metabolic phenotype and hence to disease risk^3,9,10^. Because the kidneys directly impact circulating metabolite levels, declining renal function leads to the retention of enormous metabolites, including microbiota-derived uremic toxins^11^. Also, some recent studies have shown that gut microbiota contributes to the gut-kidney axis on the various renal disease by affecting the tryptophan^12^, melamine^13^, and volatile organic compound metabolism^14^. Previously, we have shown that some metabolites having a carboxyl group, such as cholic acid (CA), hippuric acid, and, phenaceturic acid, are mainly produced by the microbiota and these plasma levels were greatly elevated in chronic kidney disease (CKD) model mice raised under specific pathogen-free (SPF) conditions which have normal commensal microbiota^15^. On the other hand, it was not observed in germ-free (GF) mice with renal failure (RF) induced by adenine-feeding. In addition, the renal dysfunction in GF-RF mice was more severe than that in SPF-RF mice. These findings allow us to speculate that carboxyl-containing metabolites play a pathological role in the progression and/or protection of CKD. However, the precise influence of the microbial metabolites having carboxyl groups to the renal functions is obscure. Thus, comprehensive, simultaneous analyses of carboxyl-containing compounds can provide a greater understanding of the relationship between CKD pathology and gut microbiota.

Liquid chromatography/tandem mass spectrometry (LC-MS/MS) is becoming a standard tool for metabolomics study because it offers high sensitivity and selectivity as well as provides structural information useful for compound identification^16,17^. A large number of microbiota-related uremic solutes have been identified by targeted or nontargeted metabolomics conducted on both animal models and patients with CKD^18–21^. However, the development of high-coverage method with accurate quantification is challenging because the concentration and physical properties of metabolites are diverse. In addition, matrix effects caused by coexisting components often loses the accuracy and reliability of analytes and affects the result.

To increase coverage and improve the accuracy and precision, chemical isotope labeling (CIL) approaches has been reported^22–25^. In addition to the high sensitivity by proton affinity group based on the group, matrix effect could be canceled for the simultaneous ionization processing both of light reagent derivative and heavy one at the same time. Accordingly, the differential semi-quantitative analysis of metabolites for two comparative samples can be performed^22^. CIL also give the more retention in reversed-phase LC which is useful for chromatographic separation. Previously, there are various CIL reagents have been reported^26–31^. However, these reagents are relatively expensive and time-consuming for reaction, which makes it difficult to use them in routine analyses. Accordingly, the development of a rapid, inexpensive, and high-throughput method is needed for the biological and physiological experiments need many samples.

**Figure 1:**
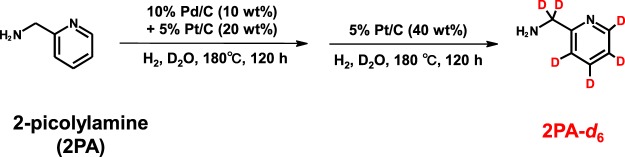
Synthesis of 2-picolylamine-d6 from 2-picolylamine.

**Figure 2:**
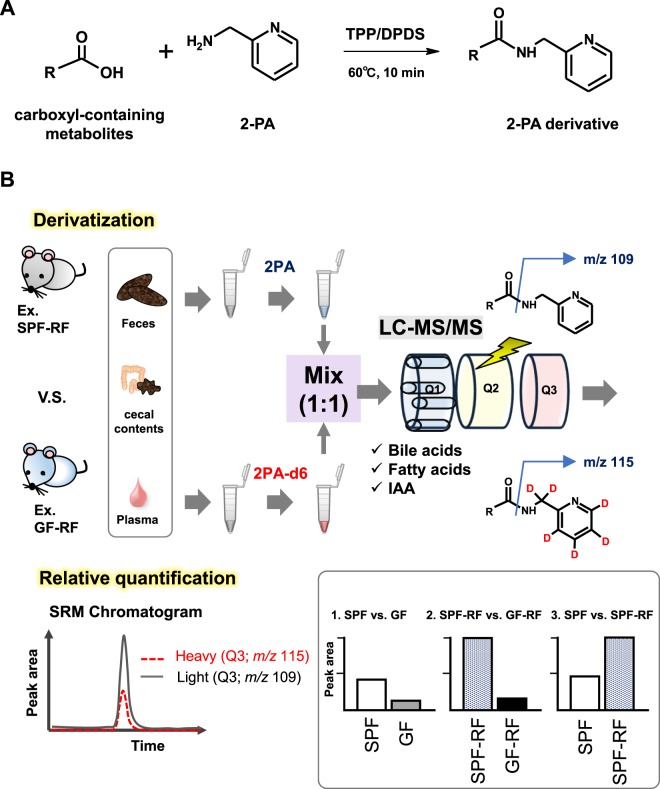
(**A**) 2PA derivatization reaction of carboxylic acid-containing metabolites. TPP: triphenylphosphine, DPDS: 2,2′-dipyridyl disulfide (**B**) Workflow of differential analysis of carboxyl-containing metabolites for plasma, feces, and cecal contents from 4 groups of mice.

In this study, to perform the comprehensive and accurate analysis of carboxyl-containing metabolites for plasma and intestinal samples in CKD model mice with or without the conditions of germ-free, we developed a CIL-LC-MS/MS method for carboxylic acid tagging with 2-picolylamine (2PA) and 2-picolylamine-*d*_6_ (2PA-d6). One of the significances of this approach is 2PA-d6 can easily synthesize through only two steps by inexpensive reagents (Fig. 1) in first. Additionally, 2PA derivatization reaction proceeds a*s* short as 10 min (Fig. 2A). To confirm the performance and applicability of the developed method for biological samples, we applied the analysis of bile acids, fatty acids, and IAA in plasma samples. In next, the accuracy of this method was compared with the absolute quantitative methods due to analysis of propionic acid (C3:0) and butyric acid (C4:0). Finally, to clarify the impact of microbiota in CKD, we compared carboxyl-containing metabolites levels in CKD model mice raised under GF conditions (GF-RF) or a normal microbiota (SPF-RF) (Fig. 2B). The developed CIL-LC-MS/MS-based approach was successfully used for comprehensive relative quantification of carboxyl-containing metabolites in plasma, feces, and cecal contents from mice and suggested that the novel features related to the microbiome and CKD pathology.

## Results

### Selection of derivatization reagents and synthesis of 2PA-d6

A simple, rapid, and inexpensive CIL-LC-MS/MS method for comprehensive profiling of carboxyl-containing metabolites could be useful for the analysis of biological and clinical samples. Because 2PA derivatization has been successfully applied for the analyses of carboxylic acids^32–34^, 2PA and its labeled compound, 2PA-d6 were chosen as derivatization reagents. Sajiki *et al*. have reported that post-synthetic H–D exchange methods for various organic compounds in D_2_O under an atmosphere of H_2_ in the presence of palladium-on-carbon (Pd/C) and platinum-on-carbon (Pt/C)^35,36^, and we applied it for the synthesis of 2PA-d6. As a result, we could complete the second step reaction (Fig. 1), the combination of Pd/C and Pt/C and subsequent the reaction with Pt/C.

### LC optimization

The chromatographic separation is critical for the analysis of many of the structural isomers of fatty acids and bile acids. Separation efficiency was evaluated under varying ionic strength, pH, and elution gradient conditions using some SCFA as representative isomers. As a result, addition of 0.1% acetic acid (v/v) in mobile phase B provided a shaper symmetric peak and enhanced resolution. In other, 0.3% of acetic acid addition or 0.1% of formic acid addition were not effective for the retention and separation for isomers such as isobutyric acid and butyric acid (Supplementary Fig. S1).

### MS/MS optimization

2PA derivatization have a highly ESI-favorable moiety, and reacts easily with carboxylic acids to form the amide derivatives^32^. The amide group is well-known fragmentable moieties in low-energy collision induced dissociation and therefore, are expected to generate a characteristic product ion suitable for the SRM detection. As shown in Supplementary Table S1, the 2PA-derivatives also provided intense protonated molecules as base peaks in the positive ESI-MS. Using these ions as the precursor ions, the most abundant product ions were detected at *m/z* 109 for 2PA or *m/z* 115 for 2PA-d6, except for the propanoic acid-*d*_5_ and butyric acid-*d*_7_ derivatives, whose product ions were [2PA (C_5_H_4_NCH_2_NH_2_) + D]^+^ at *m/z* 110 similar to the previous report^34^. Compared to the peak intensities of intact propanoic acid and butyric acid in the negative-ion mode, the 2PA-derivatives yielded the highest ionization efficiency approximately 100-fold times sensitivity (Supplementary Fig. S2).

### Evaluation of relative quantification of carboxyl-containing metabolites from standard-mixture and pooled plasma and QC plasma

In processing the method development, we recognized that the much leaching of C3:0 and C4:0 from disposable plastic tubes^37,38^. As a result of testing many types of tubes (Supplementary Fig. S3), we finally selected the siliconized-plastic tube, that elute less C3:0 and C4:0 than others, as a container for our procedure.

**Figure 3:**
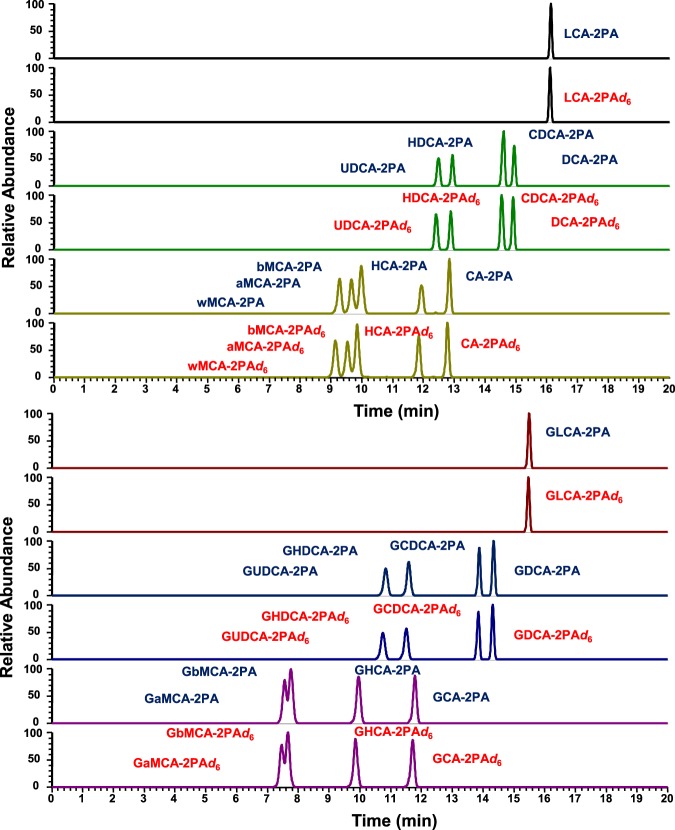
Selected reaction monitoring (SRM) chromatograms of mixture of 18 bile acid standards. The standard mixture was labeled with either 2PA or 2PA-d6, and 1:1 mixture of the labeled compounds was analyzed by RPLC triple-quadrupole mass spectrometry. LCA: lithocholic acid, UDCA: ursodeoxycholic acid, HDCA: hyodeoxycholic acid, CDCA: chenodeoxycholic acid, DCA: deoxycholic acid, wMCA: ω-muricholic acid, aMCA: α-muricholic acid, bMCA: β-muricholic acid, HCA: hyocholic acid, CA: cholic acid, GLCA: glycolithocholic acid, GUDCA: glycoursodeoxycholic acid, GHDCA: glycohyodeoxycholic acid, GCDCA: glycochenodeoxycholic acid, GDCA: glycodeoxycholic acid, GaMCA: glyco-α-muricholic acid, GbMCA: glyco-β-muricholic acid, GHCA: glycohyocholic acid, GCA: glycocholic acid.

In next, to confirm the quantitative performance of the method, we prepared an equal amount of each samples labeled with light (2PA) or heavy (2PA-d6) labeling reagents and analyzed them as mixture. For testing, five different samples (standard-mixture solutions, human plasma, mouse plasma, human QC plasma, and mouse QC plasma) were used. The detected peak area ratios of each 2PA-d6/2PA-labeled metabolites were investigated, and three independent samples were prepared for each group and analyzed in triplicate. Typical SRM chromatograms were shown in Fig. 3 (of bile acids) and Supplementary Fig. S4 (of fatty acids and IAA). The calculated peak area ratios were summarized in Table 1. However, some bile acids in plasma could not be detected due to low endogenous concentrations, the standard-mixture solutions and both QC plasma made with addition of standards gave peak pairs with almost equally peak area. In addition, most of the fatty acids and IAA also provided the equal ratio. In our approach, because the almost 2PA- and 2PA-d6 derivatives were detected at the same retention times (Figs. 3 and S4), the matrix effect are probably canceled. Accordingly, the errors from theoretical values might be caused by isotopic effect of 6 of deuterium atoms. In summary, this fundamental experiment showed that the quantitative accuracy of the developed method and usefulness as an analytical platform for carboxyl-containing metabolites.

**Table 1:** Results of peak area ratio (2PA-d6/ 2PA) (n = 3; mean ± standard deviation, %).

Analyte	*m/z* (2PA)	*m/z* (2PA-d6)	Retention Time (min)	Std. mixture	Human plasma	Mouse plasma	QC plasma (human)	QC plasma (mouse)
Propanoic acid	165.1	171.1	2.5	0.95 ± 0.11	0.92 ± 0.04	0.98 ± 0.04	0.88 ± 0.04	0.87 ± 0.01
Butyric acid	179.1	185.1	5.1	0.92 ± 0.13	0.87 ± 0.04	1.04 ± 0.11	0.83 ± 0.07	0.88 ± 0.02
Indole-3-acetic acid	266.1	272.2	11.6	0.92 ± 0.16	1.33 ± 0.13	1.37 ± 0.10	1.25 ± 0.17	1.16 ± 0.15
Lauric acid	291.1	297.2	18.6	0.99 ± 0.03	0.96 ± 0.03	1.29 ± 0.18	0.91 ± 0.12	1.02 ± 0.09
Stearidonic acid	367.3	373.1	19.9	1.27 ± 0.07	1.18 ± 0.02	1.09 ± 0.14	1.17 ± 0.13	1.22 ± 0.25
Eicosapentaenoic acid	393.2	399.3	21.1	1.37 ± 0.06	1.09 ± 0.03	0.99 ± 0.17	1.10 ± 0.17	1.14 ± 0.30
α-Linolenic acid	369.3	375.2	21.2	1.37 ± 0.04	1.12 ± 0.05	0.94 ± 0.15	1.12 ± 0.18	1.10 ± 0.25
Myristic acid	319.3	325.3	21.3	0.96 ± 0.05	1.03 ± 0.12	0.95 ± 0.11	0.84 ± 0.19	0.80 ± 0.19
γ-Linolenic acid	369.3	375.2	21.5	1.28 ± 0.05	1.08 ± 0.03	0.89 ± 0.16	1.06 ± 0.18	1.06 ± 0.27
Palmitoleic acid	345.3	351.2	22.0	1.46 ± 0.03	1.18 ± 0.05	1.04 ± 0.14	1.19 ± 0.22	1.20 ± 0.26
Docosahexaenoic acid	419.3	425.4	22.5	1.20 ± 0.06	0.84 ± 0.06	0.74 ± 0.06	0.85 ± 0.17	0.86 ± 0.27
Arachidonic acid	395.3	401.3	22.8	1.21 ± 0.06	1.10 ± 0.04	0.97 ± 0.06	1.11 ± 0.25	1.12 ± 0.35
Linoleic acid	371.3	377.3	22.9	1.05 ± 0.03	0.93 ± 0.04	0.90 ± 0.11	0.93 ± 0.20	0.91 ± 0.24
Docosapentaenoic acid	421.3	427.4	23.3	1.06 ± 0.07	0.78 ± 0.06	0.74 ± 0.13	0.78 ± 0.20	0.77 ± 0.26
Dihomo-γ-Linolenic acid	397.3	403.4	24.0	1.46 ± 0.03	1.10 ± 0.09	0.88 ± 0.08	1.13 ± 0.28	1.06 ± 0.35
Palmitic acid	345.3	351.2	24.4	0.96 ± 0.10	0.90 ± 0.09	0.78 ± 0.07	0.92 ± 0.27	0.81 ± 0.18
Adrenic acid	423.4	429.4	25.0	0.92 ± 0.12	0.69 ± 0.08	0.61 ± 0.10	0.70 ± 0.19	0.63 ± 0.24
Oleic acid	373.1	379.4	25.1	1.11 ± 0.10	0.79 ± 0.06	0.71 ± 0.11	0.82 ± 0.22	0.74 ± 0.21
Stearic acid	375.3	381.4	27.6	1.04 ± 0.07	1.13 ± 0.29	0.83 ± 0.07	1.16 ± 0.43	0.91 ± 0.24
Arachidic acid	403.4	409.4	29.6	1.39 ± 0.16	1.20 ± 0.27	0.83 ± 0.04	1.26 ± 0.45	0.94 ± 0.23
Nervonic acid	457.4	463.5	30.3	1.02 ± 0.08	0.72 ± 0.09	0.60 ± 0.11	0.71 ± 0.18	0.62 ± 0.15
Lignoceric acid	459.4	465.5	31.1	1.20 ± 0.28	0.75 ± 0.28	0.82 ± 0.04	0.88 ± 0.29	0.79 ± 0.11
Glyco-α/β-muricholic acid	556.6	562.6	7.3	0.88 ± 0.04	ND	ND	0.82 ± 0.06	0.88 ± 0.03
ω-muricholic acid	499.5	505.5	8.7	0.91 ± 0.03	ND	ND	0.84 ± 0.06	0.85 ± 0.01
α-muricholic acid	499.5	505.5	9.1	1.00 ± 0.05	ND	ND	0.88 ± 0.04	0.87 ± 0.04
β-muricholic acid	499.5	505.5	9.4	0.90 ± 0.03	ND	0.82 ± 0.12	0.85 ± 0.02	0.83 ± 0.04
Glycohyocholic acid	556.6	562.6	9.4	0.95 ± 0.05	ND	ND	0.97 ± 0.06	0.89 ± 0.07
Glycoursodeoxycholic acid	540.6	546.6	11.1	1.04 ± 0.06	ND	ND	0.90 ± 0.12	0.93 ± 0.02
Glycohyodeoxycholic acid	540.6	546.6	11.1	1.01 ± 0.04	ND	ND	0.91 ± 0.12	0.93 ± 0.02
Glycocholic acid	556.6	562.6	11.3	1.04 ± 0.05	1.24 ± 0.31	ND	1.05 ± 0.13	1.00 ± 0.04
Hyocholic acid	499.5	505.5	11.5	0.91 ± 0.04	ND	ND	0.90 ± 0.10	0.88 ± 0.04
Ursodeoxycholic acid	483.5	489.5	12.1	0.95 ± 0.04	0.95 ± 0.11	ND	0.86 ± 0.04	0.87 ± 0.01
Cholic acid	499.5	505.5	12.5	0.96 ± 0.04	0.87 ± 0.06	0.79 ± 0.05	0.84 ± 0.03	0.89 ± 0.04
Hyodeoxycholic acid	483.5	489.5	12.6	0.84 ± 0.01	0.92 ± 0.16	ND	0.82 ± 0.03	0.84 ± 0.03
Glycochenodeoxycholic acid	540.6	546.6	14.1	1.08 ± 0.15	0.55 ± 0.20	ND	0.87 ± 0.10	0.71 ± 0.12
Glycodeoxycholic acid	540.6	546.6	14.1	0.98 ± 0.04	0.93 ± 0.04	ND	0.91 ± 0.07	0.93 ± 0.03
Chenodeoxycholic acid	483.5	489.5	14.1	0.97 ± 0.05	0.88 ± 0.03	ND	0.87 ± 0.03	0.91 ± 0.02
Deoxycholic acid	483.5	489.5	14.8	0.97 ± 0.04	0.90 ± 0.06	0.99 ± 0.10	0.92 ± 0.06	0.93 ± 0.04
Glycolithocholic acid	524.6	530.6	15.3	1.03 ± 0.06	0.82 ± 0.10	ND	0.86 ± 0.07	0.89 ± 0.10
Lithocholic acid	467.5	473.5	16.0	0.90 ± 0.03	0.91 ± 0.04	ND	0.89 ± 0.08	1.00 ± 0.14

ND; Non-detected.

### Comparison of absolute quantification method and CIL LC–MS/MS methods

For verification of the developed semi-quantitative method, the comparison with absolute quantitation was performed. In first, to obtain the real concentrations of propanoic acid and butyric acid in pooled plasma samples, we performed absolute quantification based on standard calibration curves. Stable isotopes of propanoic acid-*d*_5_ and butyric acid-*d*_7_ were used as ISs and calibration curves were plotted using seven calibration concentrations. Correlation coefficients of two analytes were above 0.99 for both analytes, indicating good linearity across the testing range (Supplementary Table S2). A both in human pooled plasma and mouse pooled plasma, the all precision and accuracy values satisfied all criterion for all measured concentrations (Supplementary Table S3). These data suggested that the 2PA-labeling based method could also be applicable for absolute quantification.

**Figure 4:**
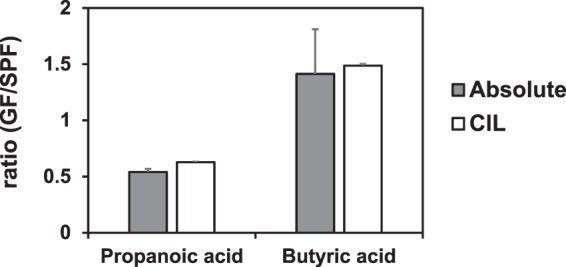
GF-to-SPF ratios of propanoic acid and butyric acid determined by the 2PA derivatization LC-MS/MS method (Absolute) and by the CIL-LC-MS/MS method (CIL). Bars indicate means ± SD. Gray bars indicate the ratio was obtained from absolute quantitation method; Three independent plasma samples were prepared for each group mice and separately labeled by 2PA. GF/SPF ratios of propanoic acid and butyric acid were calculated by mean of the absolute concentrations. White bars indicate the ratio was obtained from CIL-LC-MS/MS method; The plasma samples were obtained by combining equal volume (n = 3) of each group mice. The pooled plasma was labeled with 2PA (for SPF) and 2PA-d6 (for GF), respectively. Then labeled samples were mixed (1:1) and subjected to the differential analysis. GF/SPF ratios of propanoic acid and butyric acid were calculated by the mean of ion peak area in triplicate measurements.

Next, to confirm the accuracy of relative quantitative performance of the developed method, we analyzed two SCFAs, propanoic acid and butyric acid in plasma by both absolute quantification and relative quantification, and compared the two results by two GF-to-SPF ratios of two SCFAs. As shown in Fig. 4, the GF-to-SPF ratios of C3:0 and C4:0 by semi-quantitative method were equal to the results by absolute quantitation. This result suggested that the semi-quantitative approach provide the accurate metabolite ratios.

### Profiling of carboxyl-containing metabolites in plasma, feces, and cecal contents from SPF, SPF-RF, GF, and GF-RF mice

**Figure 5:**
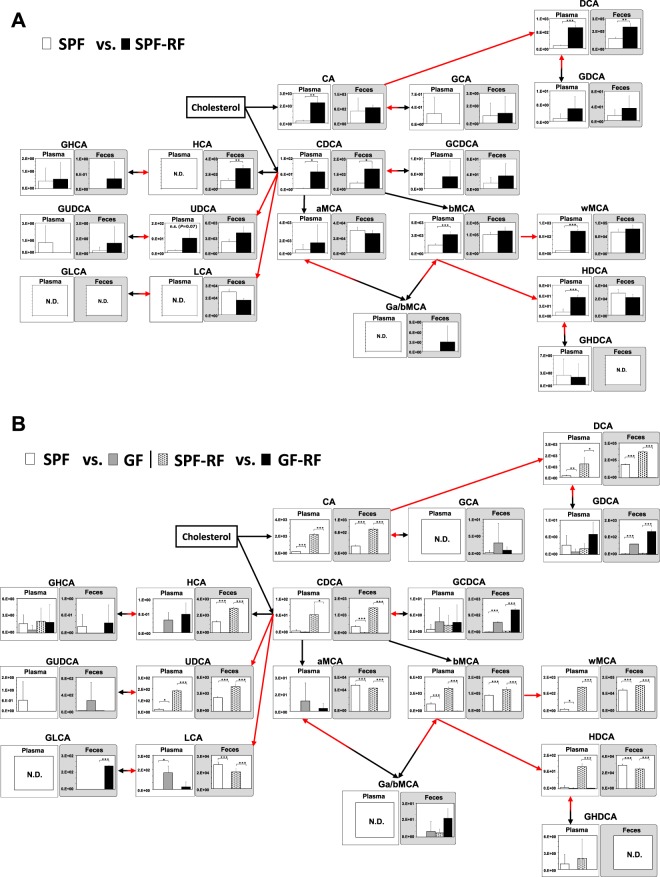
Reconstruction of bile acid metabolic pathways from 18 bile acids. Relative quantitative analysis of the bile acids in plasma and feces between (**A**) SPF and SPF-RF groups. (**B**) SPF and GF groups, SPF-RF and GF-RF groups. Metabolic pathways are involved in host and microbiota as indicated by black arrows and red arrows, respectively. Bars indicate means ± SD of ion peak area in triplicate measurements. *P < 0.05, **P < 0.01, ***P < 0.001 compared between indicated groups (Student’s t test). The pooled samples were prepared from each groups of mice; N = 4 in SPF control (SPF) and GF control (GF), and N = 3 in SPF renal failure (SPF-RF), and N = 5 GF renal failure (GF-RF) mice.

In this study, we focused on microbiota-derived carboxyl-containing metabolites, such as SCFAs, secondary bile acids, and IAA, because they have critical functions for regulating many biological processes^9,39–41^. Regarding the role of renal disease, SCFAs have been shown to have renoprotective effects on the local and systemic inflammation in CKD and acute kidney injury^42,43^. Thus, in order to clarify the impact of microbiota in CKD, we prepared the 4 groups of mice (SPF, SPF-RF, GF, and GF-RF) and subsequently, measured 40 carboxyl-containing metabolites in plasma, feces, and cecal contents using a developed CIL-LC-MS/MS method. Hematoxylin and eosin (H&E) and Masson’s trichrome staining indicated that renal inflammation in GF-RF were more severe than that SPF-RF, although the renal fibrosis in GF-RF was less severe than that in SPF-RF (Supplementary Fig. S5). The clinical parameters (*e.g*. BUN, plasma creatinine) has been described previously^15^. Reconstruction of bile acid metabolic pathways from 18 bile acids data is shown in Fig. 5 (plasma and feces) and Supplementary Fig. S6 (cecal contents). Although the plasma levels of CA, chenodeoxycholic acid (CDCA), deoxycholic acid, β-muricholic acid, ω-muricholic acid, and hyodeoxycholic acid were greatly elevated in SPF-RF compared with those in SPF (Fig. 5A), they were not or nearly undetectable in GF groups (Fig. 5B). In addition, ursodeoxycholic acid and lithocholic acid were not or nearly undetectable in feces from GF and GF-RF (Fig. 5B). Accordingly, the absence of microbiota resulted in the greatly reduce of these bile acids from GF mice, indicating that these bile acids mostly originated from microbiota metabolism. Although CA and CDCA which are synthesized in the host liver from cholesterol were not detected in GF mouse group, the glycine conjugate (*e.g*. glycochenodeoxycholic acid) were elevated in the feces (Fig. 5B) and cecal contents (Supplementary Fig. S6). This suggests that glycine-conjugated bile acids were not de-conjugated by intestinal bacteria, hence most of them were expected to be present in the intestine as a conjugate. The alteration of bile acid composition how influence on the renal functions is currently unknown, but it has been reported that secondary bile acid-producing bacteria contribute to the homeostasis of glucose and lipid metabolism^44^. A further study of the precise effect of bile acids in the blood and intestine on CKD should be conducted.

**Figure 6:**
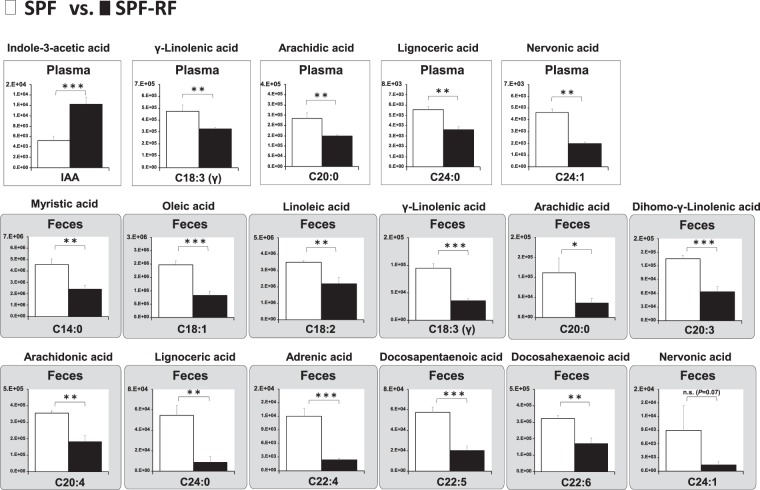
A panel of statistically significant fatty acids and IAA in plasma and feces between SPF and SPF-RF groups. Bars indicate means ± SD of ion peak area in triplicate measurements. *P < 0.05, **P < 0.01, ***P < 0.001 compared between indicated groups (Student’s t test).

**Figure 7:**
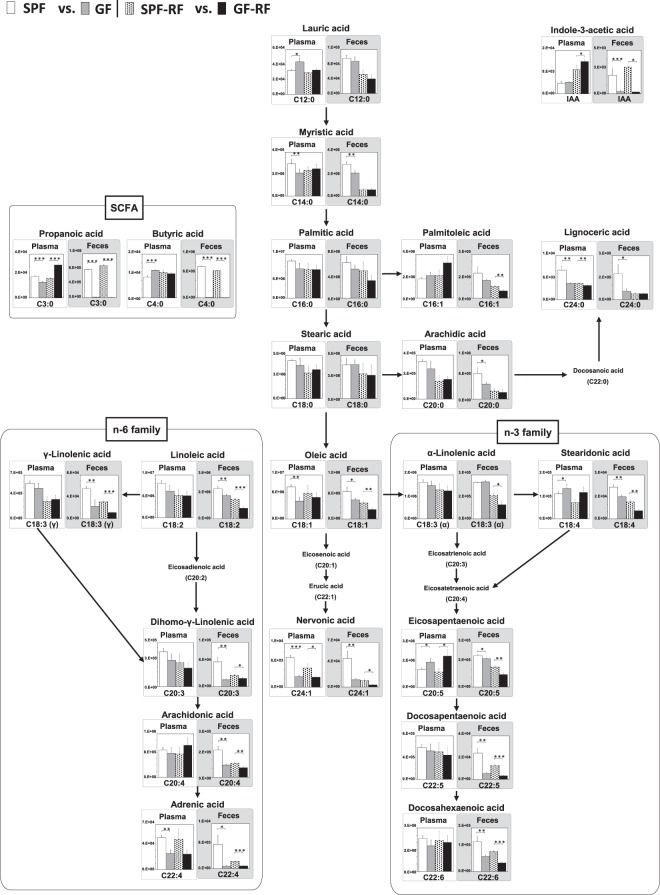
Reconstruction of integrate metabolic pathways from 19 fatty acids, SCFA, and IAA. Relative quantitative analysis of the fatty acids and IAA in plasma and feces between SPF and GF groups, SPF-RF and GF-RF groups. Bars indicate means ± SD of ion peak area in triplicate measurements. *P < 0.05, **P < 0.01, ***P < 0.001 compared between indicated groups (Student’s t test).

Considering bile acids are secreted into the duodenum to facilitate dietary fat digestion and absorption, it can be presumed that drastic change of bile acids in GF mice affect fatty acid metabolism. Further, Kindt *et al*. clarify that the gut microbiota promotes hepatic fatty acid desaturation and elongation in mice^45^. Thus, we hypothesized that GF-RF mice differ from SPF-RF mice in their fatty acid metabolism, leading to the progression of CKD. Also, IAA is uremic solute produced from the dietary tryptophan by gut microbiota, it has been reported to an influence on the host lipid metabolism, via aryl hydrocarbon receptor (AhR) pathway^41,46–48^.To address this point, we measured IAA and 21 fatty acids, including SCFA, saturated fatty acid, monounsaturated fatty acids, long-chain omega-6 (n-6), and omega-3 (n-3) polyunsaturated fatty acids (PUFA) using a developed method. There was no fatty acid that elevated in SPF-RF plasma compared with those in SPF. On the contrary, a significant decrease of γ-linolenic acid (C18:3 n-6), arachidic acid (C20:0), lignoceric acid (C24:0), and nervonic acid (C24:1) was detected in SPF-RF as compared to controls (Fig. 6). The reason for this decreasing is currently unknown, but the result of γ-linolenic acid is in agreement with the previous study, that was conducted on CKD patients plasma^49^. Also, the fecal levels of myristic acid (C14:0), oleic acid (C18:1), linoleic acid (C18:2), C18:3 n-6, C20:0, dihomo-γ-linolenic acid (C20:3), arachidonic acid (C20:4), C24:0, adrenic acid (C22:4), docosapentaenoic acid (C22:5), and docosahexaenoic acid (C22:6) were significantly lower in SPF-RF than in SPF (Fig. 6). Notably, these fecal fatty acids and palmitoleic acid (C16:1) were also lower in the GF groups than in the SPF groups, and hence GF-RF mice were the lowest levels among 4 groups (Fig. 7). These results indicating that fatty acid levels in the intestine were affected not only by renal function but also by gut microbiota. This is further supported by the results of cecal contents (Supplementary Fig. S7). The plasma levels of C14:0, C18:1, C24:1, and C22:4 were significantly lower in GF than in SPF. In contrast, lauric acid (C12:0), stearidonic acid (C18:4), and eicosapentaenoic acid (C20:5) were higher in GF than in SPF (Fig. 7). These findings suggest that the absence of microbiota which has an important role in desaturation and elongation of fatty acid caused substantial changes in plasma fatty acid profile. Because a certain gut lactic acid bacterium plays a polyunsaturated fatty acid-saturation metabolism, a part of this result could be due to the absence of these microbes^4^.

SCFA and IAA are produced from the dietary fibre and tryptophan by gut microbiota, respectively^39,47^. As SCFA and IAA are a microbiota-associated metabolite, the plasma C3:0, C4:0, and IAA levels are considered to be governed by microbiota-derived production. However, the present findings revealed that the absence of microbiota did not significant affect plasma these levels in the GF groups, suggesting that host-derived production accounted for a much larger population of the total amounts (Fig. 7). In our previously report that was conducted on CE-TOF MS-based analysis, the plasma C4:0 level was below the limits of detection^15^. Therefore, the developed CIL-LC-MS based approach provided a more high-sensitive platform for analyses SCFA. In agreement with the previous our studies, C3:0 and C4:0 were nearly undetectable in the feces of GF and GF-RF, suggesting that intestinal SCFA production was ceased in both the GF groups. Likewise, fecal and cecal contents IAA levels were significantly lower in the GF groups than in the SPF groups (Figs. 7 and S7).

## Discussion

In this study, to overcome some limitations of current metabolomics methods for carboxyl-containing metabolites, we developed a novel CIL-LC-MS/MS method using the 2PA and its isotopologue as derivatization reagents. This method was a reasonable approach for analyzing the carboxyl submetabolome profiling with many samples, because not only 2PA-d6 could easily be synthesized with inexpensive reagents, but also derivatization reaction was simple and quick. The quantitative performance of it was verified by many of experiments. In all result, this semi-quantitative method provided the high accuracy and high sensitivity as well. In addition, our approach is not affected by matrix effect and favorable due to short sample preparation time and fewer analysis times with mixing different samples.

More recently, several microbiota strains have been shown to be responsible for the production of harmful uremic toxins (*e.g*. trimethylamine-*N*-oxide; indoxyl sulfate). These uremic toxins are an independent predictor of mortality in CKD patients^50^. Further, we have revealed that phenyl sulfate, one of the microbiota-derived uremic toxins, contribute to albuminuria in diabetic kidney disease^51^. However, in the previous another study, we observed the paradoxical finding that the absence of microbiota exacerbated renal damage in adenine-induced GF-RF mice, despite the attenuating accumulation of such harmful uremic solutes^15^. Also, it has been reported that GF mice had enhanced renal structural injury and functional decline following ischemia-reperfusion compared with conventional mice^52^. These findings suggest that some microbiota-derived metabolites have potential protective effects against CKD progression and share a mutually beneficial relationship with their host. There is increasing evidence that microbiota-derived SCFAs have a renoprotective effect^39^, however, the other carboxyl-containing metabolites how influence on CKD pathology has been little investigated. Using the developed method, we revealed that most bile acid levels in plasma were significantly elevated in SPF-RF mice compared to control mice, as well as the intestinal level of CDCA, HCA, and DCA were increased (Fig. 5A). Although IAA, a circulating uremic toxin, was also elevated in SPF-RF plasma, some fatty acid levels were decreased (Fig. 6). It should be noted that the absence of microbiota caused decreasing bile acids, fatty acids, and IAA levels in the intestine (Fig. 5B, Supplementary Fig. S6, Fig. 7, and Supplementary Fig. S7). In addition, the renal failure enhanced the decrease of the intestinal level of n-3 PUFAs (Figs. 6 and 7). Interestingly, it has also been reported that bile acids, n-3 PUFA, SCFA, and IAA, which are focused in this study, play important roles in the endothelial barrier function, respectively^41,53,54^. A number of studies have demonstrated the negative effects of CKD on the gut microbiota and intestinal barrier function^18,19,55^.Recently, it was shown that the metabolome disorders in rat with CKD induced by 5/6 nephrectomy or unilateral ureteral obstruction cause decline in microbial diversity and richness, which were mediated by dysregulations of lipids, amino acids, and bile acids metabolism^18,19^. Gut dysbiosis, seen commonly in patients with CKD, caused by a decline in microbial diversity and disruption of gut barrier function^56^. The disrupt of the intestinal epithelial barrier lead to infiltration of various antigen and pro-inflammatory products into the systemic circulation, thus it facilitates the pathogenesis of inflammation in CKD^57^. Taken together, these observations indicated the decrease of intestinal bile acids, IAA, SCFA, and n-3 PUFAs caused by both renal damage and the absence of microbiota might be also contributed to the exacerbating effects on the intestinal epithelial barrier function that results in CKD progression in GF-RF mice (Supplementary Fig. S8). It was reported that treatment with 5-methoxytryptophan, which plasma levels decrease with progression of CKD, ameliorates renal injury in mouse via the anti-inflammatory and anti-fibrotic effects^58^. Thus, supplementation with diet or pre-, pro- and symbiotic approach to correct intestinal bile acids, IAA, SCFA, and n-3 PUFAs levels may also lead to potential protective effects against CKD progression by suppressing epithelial barrier disruption.

The primary limitation in this study is that the differences in the plasma metabolites between SPF and SPF-RF (or SPF-RF and GF-RF) may be attributed not only to the decline of renal clearance but also to confounding factors such as body weight, body fluid balance, cage effects^59^, and dietary intake^15^. Also, the disruption of the gastrointestinal epithelial barrier function might promote adenine absorption, that results in CKD progression. Thus, to verify our findings in the present study, additional research using other CKD models is required.

In conclusion, we suggest that monitoring a panel of microbiota-derived carboxyl-containing metabolites by the developed CIL-LC-MS/MS method would be useful for obtaining an in-depth appreciation of the compounds involves in the gut–kidney axis and physiological and pathological roles of CKD. Taking the advantages in selectivity and applicability afforded by 2PA into account, the method developed herein is capable of simultaneous analysis of multiple carboxyl-containing compounds. We hope that the outcome of the present study will contribute to a better understanding of the impact of gut microbiota on CKD progression.

## Materials and Methods

### Chemicals and reagents

CA, CDCA, and palladium-activated carbon (Pd:10%) were purchased from Sigm–Aldrich (St. Louis, MO). LC/MS-grade ammonium acetate (AcONH_4_) and acetic acid (AcOH), deoxycholic acid, lithocholic acid, IAA, C3:0, C4:0, and platinum-activated carbon (Pt:5%) were purchased from FUJIFILM Wako Pure Chemical Industries (Osaka, Japan). Ursodeoxycholic acid, glycoursodeoxycholic acid were purchased from Nacalai Tesque, Inc. (Kyoto, Japan). Glycocholic acid, glycohyocholic acid, glycochenodeoxycholic acid, glycodeoxycholic acid, glycolithocholic acid, hyocholic acid, hyodeoxycholic acid, α-muricholic acid, β-muricholic acid, ω-muricholic acid, glycohyodeoxycholic acid, glyco-α-muricholic acid, glyco-β-muricholic acid were synthesized in our laboratory using previously reported method^60^. Propanoic acid-*d*_5_ and butyric acid-*d*_7_ were obtained from Toronto Research Chemicals (North York, ON, Canada). 2-picolylamine (2PA), 2,2′-dipyridyl disulfide (DPDS) and triphenylphosphine (TPP) were obtained from Tokyo Chemical Co. (Tokyo, Japan). Deuterium oxide (99.9% isotopic purity), LC/MS-grade methanol (MeOH), 2-propanol (IPA), and acetonitrile (MeCN) were obtained from Kanto Chemical (Tokyo, Japan). Human pooled plasma and mouse plasma were obtained from Biopredic International (Rennes, France) and Rockland Immunochemicals Inc. (Limerick, PA, USA), respectively. Saturated/monounsaturated fatty acid mixture and polyunsaturated fatty acid mixture were purchased from Cayman Chemicals (Ann Arbor, MI, USA). Ultra-pure water was purified by a PURELAB ultra water purification system (Organo Co. Ltd, Tokyo). The reagents used for the derivatization were adjusted using MeCN. All stock solutions were stored at −20 °C and avoid light.

### Animal treatment and sample collection

The plasma, feces, and cecum from GF and SPF mice were collected at previous work and stored at −80 °C in our laboratory^15^. Briefly, GF and SPF IQI mice were purchased from Clea Japan (Tokyo, Japan). At 7 weeks of age, each group of mice was randomly divided into control and RF groups. For the GF and SPF control groups, normal sterilized CE-2 diet was continued for 5 weeks (GF, SPF). For the RF groups, sterilized CE-2 diet containing 0.2% adenine (Wako Pure Chemical Industries, Japan) was given for 5 weeks (GF-RF, SPF-RF). In the final 2 weeks of this 5-week period, the RF mice were given a normal diet for 2 days per week not to weaken. At the end of the study, the mice were killed under isoflurane-induced anesthesia, and blood, fresh feces, and cecum contents were collected.

### Extraction of metabolites from feces and cecal contents

10 mg of feces or contents in cecum were homogenized in 1.0 mL of MeOH for 2 min and then centrifuged at 15,000 g for 10 min. The obtained supernatants were combined followed by CIL-LC-MS analysis.

### Synthesis of 2-picolylamine-*d*_6_

2PA-d6 were synthesized from 2PA. Deuterium-labeling (H-D exchange) reaction was examined according to previously reported methods^35,61^. 2PA (540.7 mg, 5.0 mmol) and 10% Pd/C (54.0 mg, 10 wt % of the substrate) and 5% Pt/C (108.1 mg, 20 wt % of the substrate) in D_2_O (18 mL) were stirred at 180 °C in a sealed tube under H_2_ atmosphere for 120 h. After cooling, the reaction mixture was filtered through a filter (Millipore Millex-LG, 0.20 mm) to paper to remove the catalyst. The filtered catalyst was washed with D_2_O (1 mL). The filtrate and 5% Pt/C (224.9 mg, 40 wt % of the substrate) were stirred at 180 °C in a sealed tube under H_2_ atmosphere for 120 h. The filtered catalyst was washed with D_2_O (1 mL) and the filtrate was concentrated in vacuo. Next, the residue was purified by reversed-phase chromatography (H_2_O/MeOH) to yield 2PA-d6. Chromatographic separation was carried out on DualPore ODS (21 mm i.d. × 75 mm, 12 g, DPS, Tokyo, Japan) at room temperature. UV spectra were recorded on LC-Forte/R system (YMC, Kyoto, Japan). The extracts are evaporated to dryness, redissolved in MeCN. Synthesized structures were confirmed by high-resolution mass spectrometry and ^1^H NMR spectroscopy. High-resolution mass spectra were recorded on a Q Exactive (Thermo Fisher Scientific, Waltham, MA, USA). ^1^H NMR spectra were recorded on a JEOL JNM-ECA600 spectrometer. The concentration was determined by LC-UV analysis. UV spectra were recorded on Nexera UHPLC system (Shimadzu, Kyoto, Japan).

### Labeling of sample with 2PA and 2PA-d6

2PA or 2PA-d6 was used to label carboxyl-containing metabolites (Fig. 2A). The labeling condition (reaction temperature and time) were according to previous reports^32^. Briefly, for standard-mixture, 200 µL of MeCN and 10 µmol/L C3:0-*d*_5_ and C4:0-*d*_7_ mixed solution (30 µL, if absolute quantitation) as internal standards (ISs) added to the samples (20 µL) were placed into a siliconized 1.5-mL sample tube (Watson, Kobe, Japan) and 10 mM TPP in MeCN (10 µL), 10 mM DPDS in MeCN (10 µL) and, 10 mM 2PA in MeCN (10 µL) or 10 mM 2PA-d6 (10 µL) were added for derivatization. The mixture was then kept at 60 °C for ~10 min. The resulting solution was evaporated to dryness by a rotary vacuum evaporator at 40 °C. The dried sample was reconstituted with 50 µL of 50% MeOH in water. Finally, the labeled samples were mixed in equal volume before LC-MS/MS analysis.

For 20 µL aliquots of biological samples, placed into a 1.5-mL sample tube and MeCN (200 μL) was added for deproteinization. The obtained mixture was homogenized for 5 min in an ultrasonic bath. After centrifugation at 15,000 × *g* for 10 min at 4 °C, 200 µL of supernatant was collected into a new siliconized tube, followed by 2PA or 2PA-d6 derivatization and LC-MS/MS analysis in the same way as described above.

### Instrumentation and LC-MS/MS condition

The LC-MS/MS analyses were conducted on a Nexera UHPLC system (Shimadzu, Kyoto, Japan) coupled to a TSQ Quantum Vantage (Thermo Fisher Scientific, Waltham, MA, USA) triple quadrupole mass spectrometer. Chromatographic separation was carried out on Intertsil ODS-3 (2.1 mm i.d. × 100 mm, 2 μm, GL Science, Tokyo, Japan) at 45 °C. The mobile phase was 0.1% AcOH with 10 mM AcONH_4_ in water (A), and 0.1% AcOH in MeCN/IPA (v/v = 1:1) (B). The flow rate was set at 430 µL/min for analysis of fatty acids or IAA or 450 µL/min for analysis of bile acids. An elution gradient was used as follows: for analysis of fatty acids and IAA, initial elution with 5% B, followed by a linear gradient to 20% B from 1.0–10.0 min, to 60% B from 10.1–15.0 min, to 80% B from 15.1–28.0 min, and, thereafter, to 95% B at 28.1 min, held at 95%B for 32.0 min. Columns were re-equilibrated with 5% B for 3.0 min prior to the next injection. For analysis of bile acids, initial elution with 30% B, followed by a linear gradient to 40% B at 10.0 min, to 70% B from 10.1–15.0 min and, thereafter, to 95% B at 15.1 min, held at 95%B for 17.5 min. Columns were re-equilibrated with 30% B for 2.5 min prior to the next injection.

The mass spectrometer was equipped with a heated electrospray ionization (HESI) source. HESI was performed in positive ion modes. Nitrogen was used for nebulization and desolvation and argon was used as the collision gas. The capillary voltage was set at 3.0 kV. The optimum conditions were: sheath gas pressure 60 psi; auxiliary gas pressure 60 psi; vaporizer temperature 500 °C; capillary temperature 350 °C; declustering voltage −10eV, and collision gas pressure 1.5 mTorr. The selected reaction monitoring (SRM) transitions, S-lens voltage, and collision energy are shown in Supplementary Table 1. The LC-MS/MS system was controlled by Xcalibur software (Thermo Fisher Scientific, Waltham, MA, USA), which was also used to collect data.

### Preparation of calibration curve, mixed standard solution and quality control (QC) samples for analysis of C3:0 and C4:0

C3:0, C4:0 and their corresponding ISs (C3:0-*d*_5_ and C4:0-*d*_7_) were weighed accurately into volumetric flasks and then diluted separately with 50% MeOH in water, to produce stock solutions, which were stored at −20 °C. Before use, stock solutions were serially diluted with MeCN to give a mixed working solution series. Calibration standards were prepared by diluting the ISs with MeCN. QC samples were prepared from working solutions or mixed standard solutions diluted in blank mouse plasma and human plasma (v/v = 1: 9). A 20-µL sample was labeled by 2PA following the same procedure described above for sample labeling. Calibration was performed using nine-point standards across the desired quantification range. Calibration curves were constructed using analyte-to-IS peak area ratios and 1/X weighted linear regressions. All peaks were integrated automatically by the Xcalibur software.

### Evaluation of relative quantification of carboxyl-containing metabolites from standard-mixture, pooled plasma, and QC plasma

The mixture solution of 3 carboxyl compounds (bile acid, fatty acid and IAA) with different structures was used to evaluate the accuracy of the relative quantification. The mixed standard solution was combined in a single solution consisting of 19 bile acids mixture (30 µmol/L), 9 saturated-monounsaturated fatty acids mixture (3.75 µg/mL), 10 polyunsaturated fatty acids mixture (3.75 µg/mL), C3:0 (20 µmol/L), C4:0 (20 µmol/L), IAA (50 µmol/L), and stored at −20 °C. Before use, the mixed standard solution was diluted 10-fold with MeCN. QC samples were prepared from working solutions or mixed standard solutions diluted in blank mouse plasma and human plasma (v/v = 1: 9).

To prepare the 1:1 2PA-d6/2PA-labeled sample for analytical performance evaluation, 40 µL of various samples (the mixed standard solution; blank human plasma; blank mouse plasma, and QC plasma) were divided into two parts: 20 µL was labeled by 2PA-d6 and the other 20 µL was labeled by 2PA following the same procedure described above for sample labeling. After labeling and reconstruction, the labeled samples were mixed in equal volume before LC-MS/MS analysis.

### Determination of GF-to-SPF ratios of C3:0 and C4:0 in plasma

The levels of C3:0 and C4:0 in SPF mouse plasma and GF mouse plasma were determined by 2PA derivatized-LC-MS/MS method. The precision and accuracy of the absolute quantification method for C3:0 and C4:0 were confirmed by spiking two or five concentrations of the analyte compounds into mouse pooled plasma and human pooled plasma, respectively. For method validation, six samples with specified concentrations were prepared and analyzed on the same day (intra-day precision). Precision was defined as the relative standard deviation (RSD) of independent measurements, while accuracy was expressed as the relative error (RE), calculated as follows: [(found concentration – endogenous concentration)/spiked concentration – 1] × 100 (%). GF-to-SPF ratios of C3:0 and C4:0 in plasma were calculated by both absolute quantification method and CIL method.

### Relative quantitation of carboxyl-containing metabolites in plasma, feces, and cecal contents

The workflow of the experiment for profiling of carboxyl-containing metabolites is shown in Fig. 2B. The plasma, feces, and cecal contents samples were obtained by combining equal volume of each sample from 4 groups of mice, respectively: SPF control (SPF) mice (n = 4), GF control (GF) mice (n = 4), SPF-RF mice (n = 3), and GF-RF mice (n = 5). The protocol was approved by the Institutional Animal Care and Use Committee at Tohoku University. All experiments were performed in accordance with the approved protocol.

### Statistical analyses

All statistical analyses were performed using JMP software (ver. 14.1.2; SAS Institute, Cary, NC, USA). Values are presented as mean ± SD. Results were evaluated using Student’s t-test. P values of < 0.05 were statistically significant.

### Ethical approval

The protocol was approved by the Institutional Animal Care and Use Committee at Tohoku University.

## Supplementary information

Table S1–3 and Figure S1–8
