# Supplementary material for: Comprehensive and semi-quantitative analysis of carboxyl-containing metabolites related to gut microbiota on chronic kidney disease using 2-picolylamine isotopic labeling LC-MS/MS
Source: Sci Rep. 2019 Dec 13;9:19075. doi: 10.1038/s41598-019-55600-1 (PMC6910927; doi:10.1038/s41598-019-55600-1)
Supplement: Supplementary file 1 — Table S1–3 and Figure S1–8 [file 41598_2019_55600_MOESM1_ESM.pdf]

Supplementary Material

Comprehensive and semi-quantitative analysis of carboxyl-containing metabolites related to gut microbiota on chronic kidney disease using 2-picolylamine isotopic labeling LC-MS/MS

Yoshitomi Kanemitsu, Eikan Mishima, Masamitsu Maekawa, Yotaro Matsumoto, Daisuke Saigusa, Hiroaki Yamaguchi, Jiro Ogura, Hiroki Tsukamoto, Yoshihisa Tomioka, Takaaki Abe, and Nariyasu Mano

Supplementary table S1. SRM transitions and compound-dependent parameters for CIL-LC-MS/MS analyses of the analytes

Analyte (abbreviation)		M+2PA				M+2PA-d6			
		Q1 (*m/z*)	Q3 (*m/z*)	CE (V)	S-lens (V)	Q1 (*m/z*)	Q3 (*m/z*)	CE (V)	S-lens (V)
Cholic acid	(CA)	499.5	109.0	20	180	505.5	115.0	20	180
α-muricholic acid	(aMCA)	499.5	109.0	20	180	505.5	115.0	20	180
β-muricholic acid	(bMCA)	499.5	109.0	20	180	505.5	115.0	20	180
ω-muricholic acid	(wMCA)	499.5	109.0	20	180	505.5	115.0	20	180
Hyocholic acid	(HCA)	499.5	109.0	20	180	505.5	115.0	20	180
Hyodeoxycholic acid	(HDCA)	483.5	109.0	20	180	489.5	115.0	20	180
Chenodeoxycholic acid	(CDCA)	483.5	109.0	20	180	489.5	115.0	20	180
Ursodeoxycholic acid	(UDCA)	483.5	109.0	20	180	489.5	115.0	20	180
Deoxycholic acid	(DCA)	483.5	109.0	20	180	489.5	115.0	20	180
Lithocholic acid	(LCA)	467.5	109.0	20	180	473.5	115.0	20	180
Glycocholic acid	(GCA)	556.6	109.0	20	180	562.6	115.0	20	180
Glyco-α-muricholic acid	(GaMCA)	556.6	109.0	20	180	562.6	115.0	20	180
Glyco-β-muricholic acid	(GbMCA)	556.6	109.0	20	180	562.6	115.0	20	180
Glycohyocholic acid	(GHCA)	556.6	109.0	20	180	562.6	115.0	20	180
Glycohyodeoxycholic acid	(GHDCA)	540.6	109.0	20	180	546.6	115.0	20	180
Glycochenodeoxycholic acid	(GCDCA)	540.6	109.0	20	180	546.6	115.0	20	180
Glycoursodeoxycholic acid	(GUDCA)	540.6	109.0	20	180	546.6	115.0	20	180
Glycodeoxycholic acid	(GDCA)	540.6	109.0	20	180	546.6	115.0	20	180
Glycolithocholic acid	(GLCA)	524.6	109.0	20	180	530.6	115.0	20	180
Propanoic acid	(C3:0)	165.1	109.0	12	55	171.1	115.0	12	55
Propanoic acid-d_5	(C3:0-d5)	170.1	110.0	14	55	176.2	116.0	14	55
Butyric acid	(C4:0)	179.1	109.0	12	66	185.1	115.0	12	66
Butyric acid-d_7	(C4:0-d7)	186.2	110.0	14	66	192.2	116.0	14	66
Lauric acid	(C12:0)	291.1	109.0	18	120	297.2	115.0	18	120
Myristic acid	(C14:0)	319.3	109.0	18	119	325.3	115.0	18	119
Palmitic acid	(C16:0)	347.3	109.0	18	119	353.3	115.0	18	120
Palmitoleic acid	(C16:1)	345.3	109.0	18	120	351.2	115.0	18	100
Stearic acid	(C18:0)	375.3	109.0	18	145	381.4	115.0	18	147
Oleic acid	(C18:1)	373.1	109.0	18	140	379.4	115.0	18	148
Arachidic acid	(C20:0)	403.4	109.0	18	151	409.4	115.0	18	150
Lignoceric acid	(C24:0)	459.4	109.0	18	164	465.5	115.0	18	174
Nervonic acid	(C24:1)	457.4	109.0	18	180	463.5	115.0	18	190
Linoleic acid	(C18:2)	371.3	109.0	18	136	377.3	115.0	16	120
α-Linolenic acid	(C18:3 n-3)	369.3	109.0	19	90	375.2	115.0	22	100
γ-Linolenic acid	(C18:3 n-6)	369.3	109.0	19	90	375.2	115.0	22	100
Stearidonic acid	(C18:4)	367.3	109.0	18	130	373.1	115.0	18	110
Dihomo-γ-Linolenic acid	(C20:3)	397.3	109.0	18	150	403.4	115.0	18	147
Arachidonic acid	(C20:4)	395.3	109.0	18	105	401.3	115.0	18	82
Eicosapentaenoic acid	(C20:5)	393.2	109.0	18	130	399.3	115.0	18	110
Adrenic acid	(C22:4)	423.4	109.0	18	156	429.4	115.0	16	160
Docosapentaenoic acid	(C22:5)	421.3	109.0	18	152	427.4	115.0	16	150
Docosahexaenoic acid	(C22:6)	419.3	109.0	18	153	425.4	115.0	16	145
Indole-3-acetic acid	(IAA)	266.1	109.0	19	100	272.2	115.0	19	108

CE; Collison energy

Supplementary Table S2. Regression equations and Correlation coefficient of propanoic acid and butyric acid

Analyte	Linear range (µmol/L)	Regression equation	Correlation coefficient
Propanoic acid	0.1 - 100	Y = 0.04782 X + 0.0057	0.999
Butyric acid	0.1 -25	Y = 0.04494 X + 0.0142	0.992

Supplementary Table S3. Intra-day precision and accuracy.

Analyte	Spiked level (μmol/L)	Human plasma†		Mouse plasma‡	
		Accuracy (%)	Precision (%)	Accuracy (%)	Precision (%)
Propanoic acid	0.5	-10.6	6.9	-	-
	1	3.5	7.5	-1.6	2.9
	5	-2.4	3.5	-	-
	10	3.4	1.7	-4.8	2.4
	75	2.6	1.7	-	-
Butyric acid	0.5	4.0	3.2	-	-
	1	-4.5	3.4	4.8	6.1
	5	-9.8	2.4	-	-
	10	0.6	1.5	-3.9	1.7

†; propanoic acid : 2.86 μmol/L, butyric acid : 0.76 μmol/L. ‡; propanoic acid: 4.11 μmol/L, butyric acid: 1.17 μmol/L. -; unexecuted

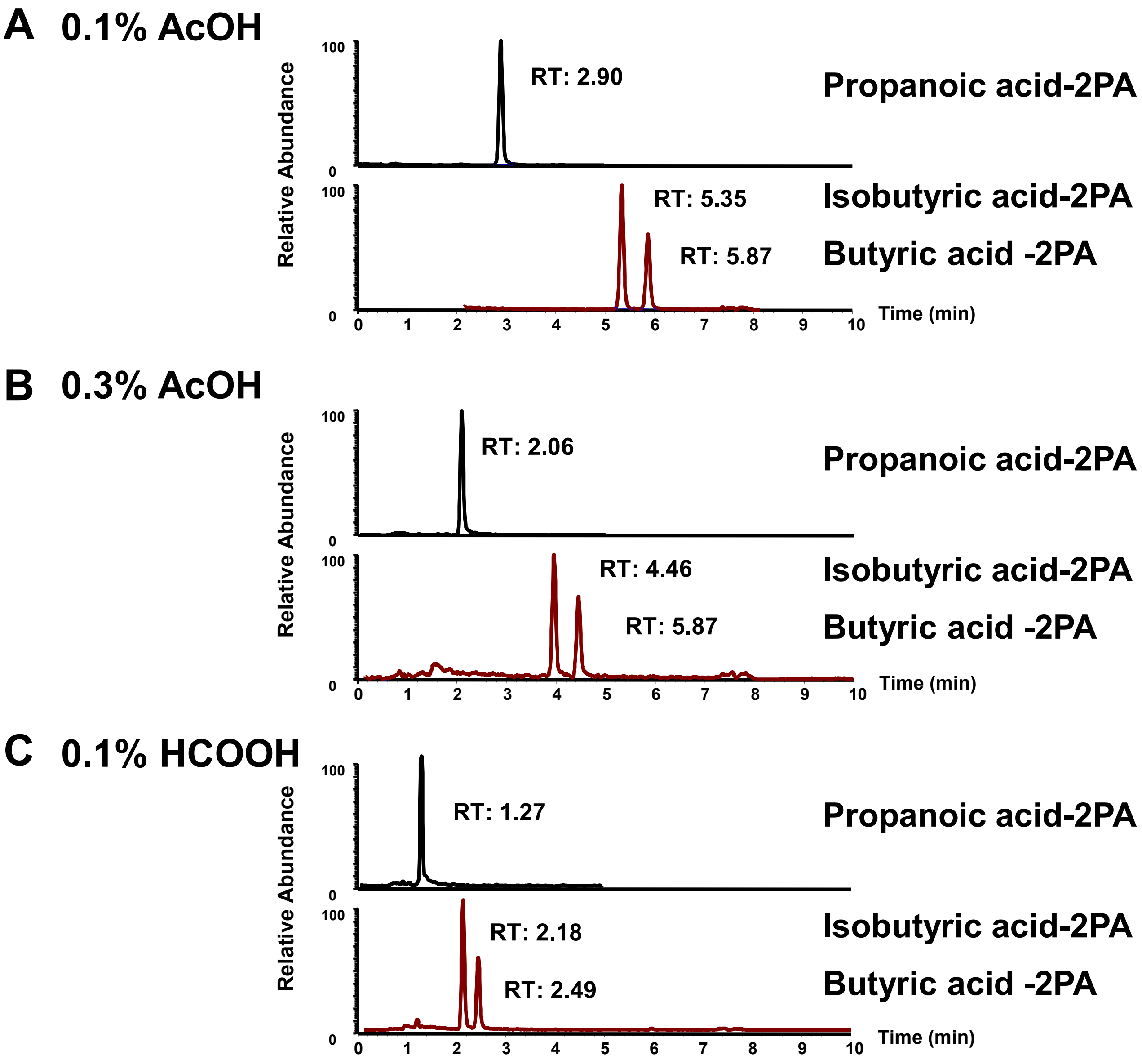

Supplementary Figure S1. Comparison of SRM chromatograms of propanoic acid and butyric acid obtained from pooled mouse plasma.
The experimental conditions were as follows: (A) 0.1% acetic acid added to the mobile phase, (B) 0.3% acetic acid added to the mobile phase, and (C) 0.1% formic acid added to the mobile phase.

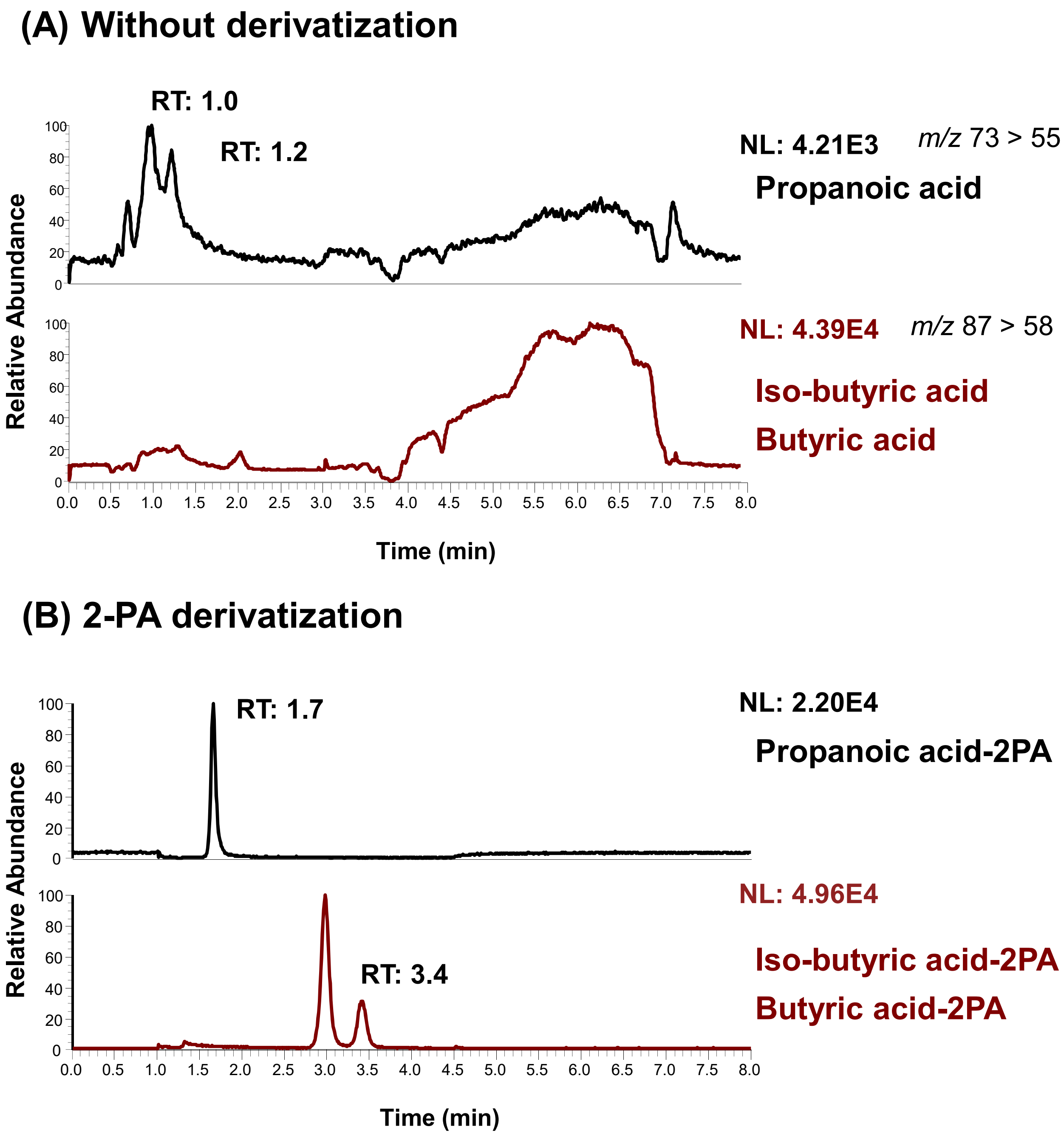

Supplementary Figure S2. SRM chromatograms and signal intensities of propanoic acid and butyric acid.

(A)The QC sample (1 μmol/L in human plasma) was analyzed without any derivatization (negative-ion mode), and (B) the same standard solutions was analyzed with the 2PA-derivatization (positive-ion mode).

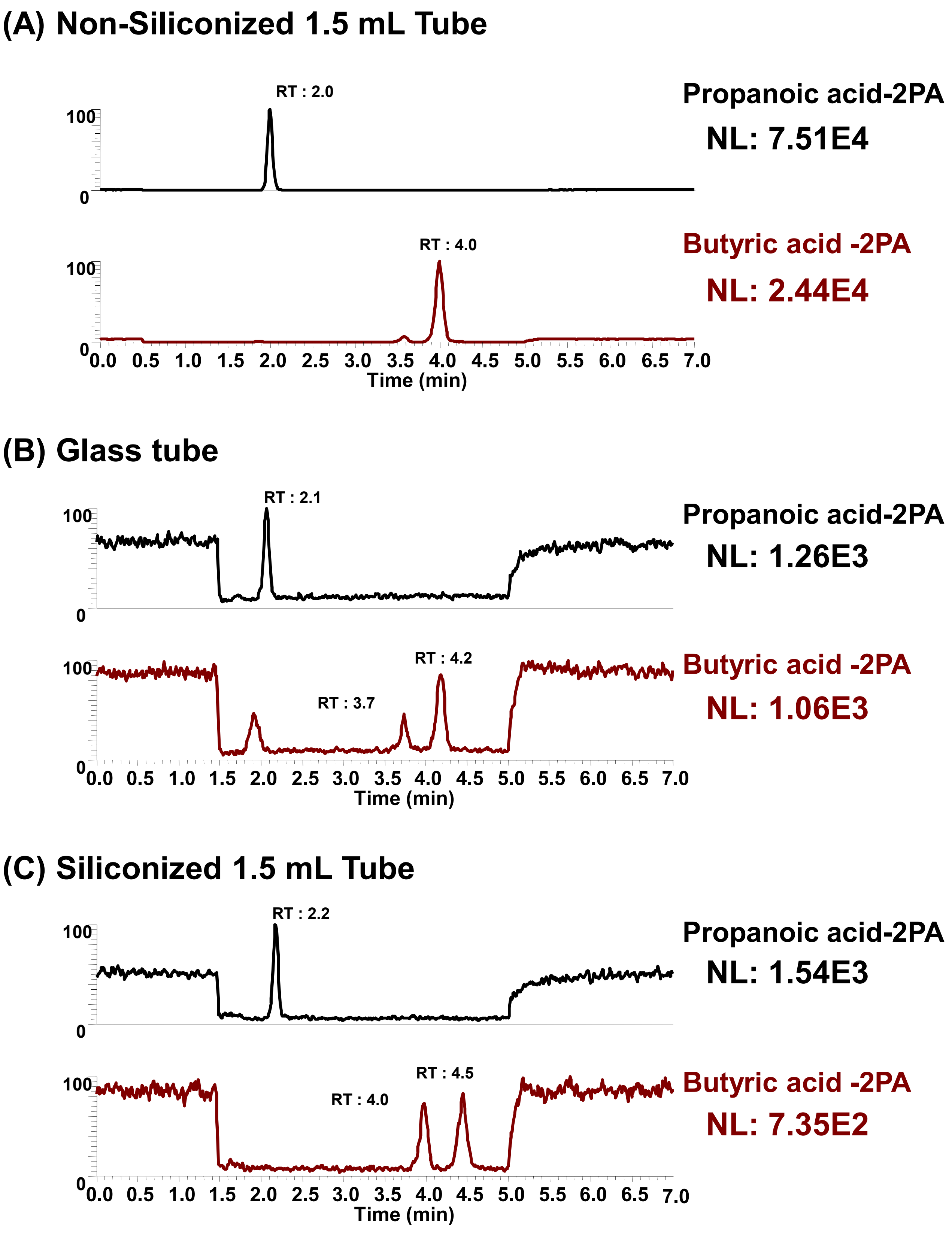

Supplementary Figure S3. SRM chromatograms and signal intensities obtained by using (A) non-siliconized plastic 1.5 mL tube, (B) glass tube, and (C) siliconized 1.5 mL tube.

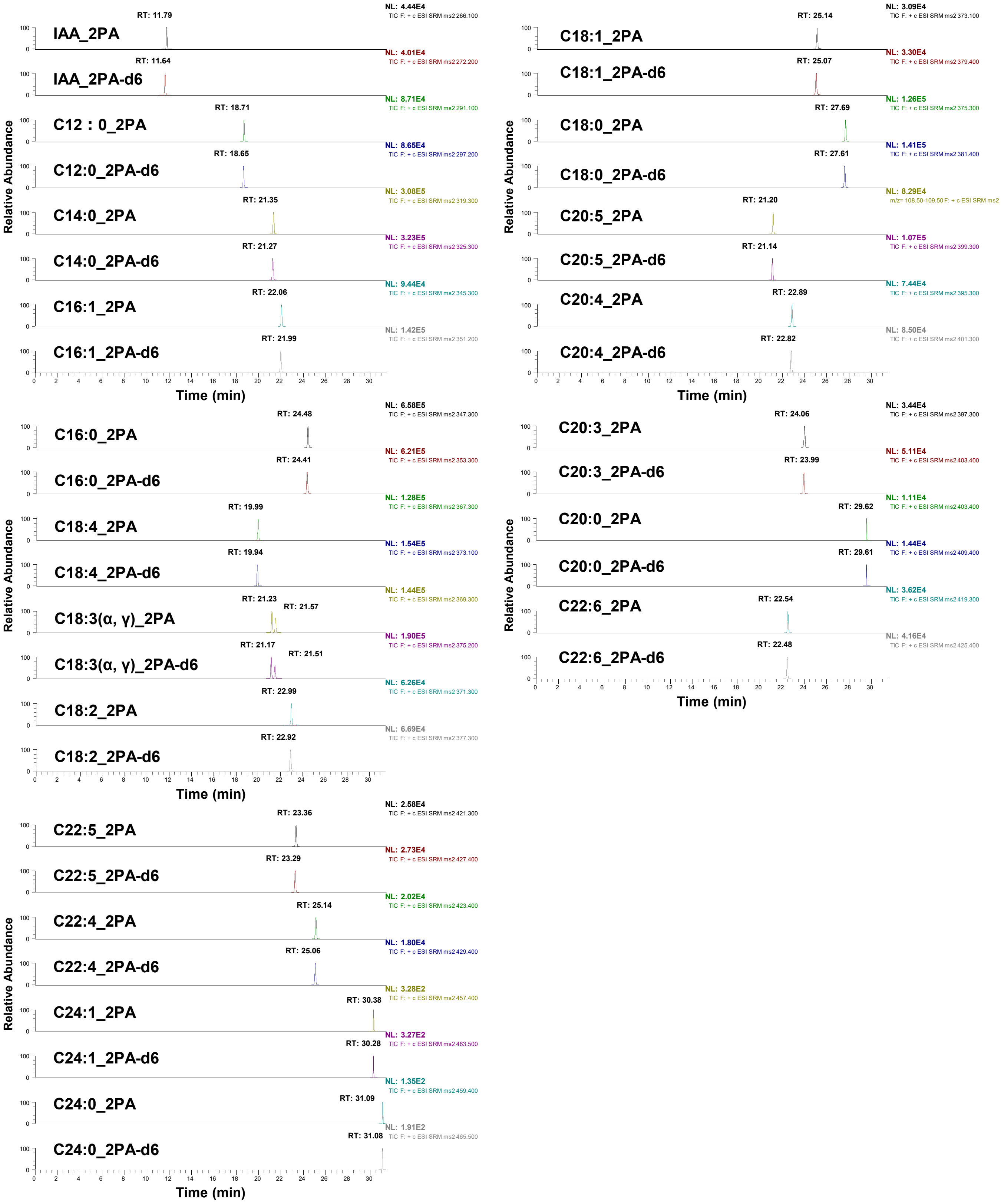

Supplementary Figure S4. SRM chromatograms of 21 fatty acids and IAA

The standard mixture was labeled with either 2PA or 2PA-d6, and 1:1 mixture of the labeled compounds was analyzed by RPLC triple-quadrupole mass spectrometry.

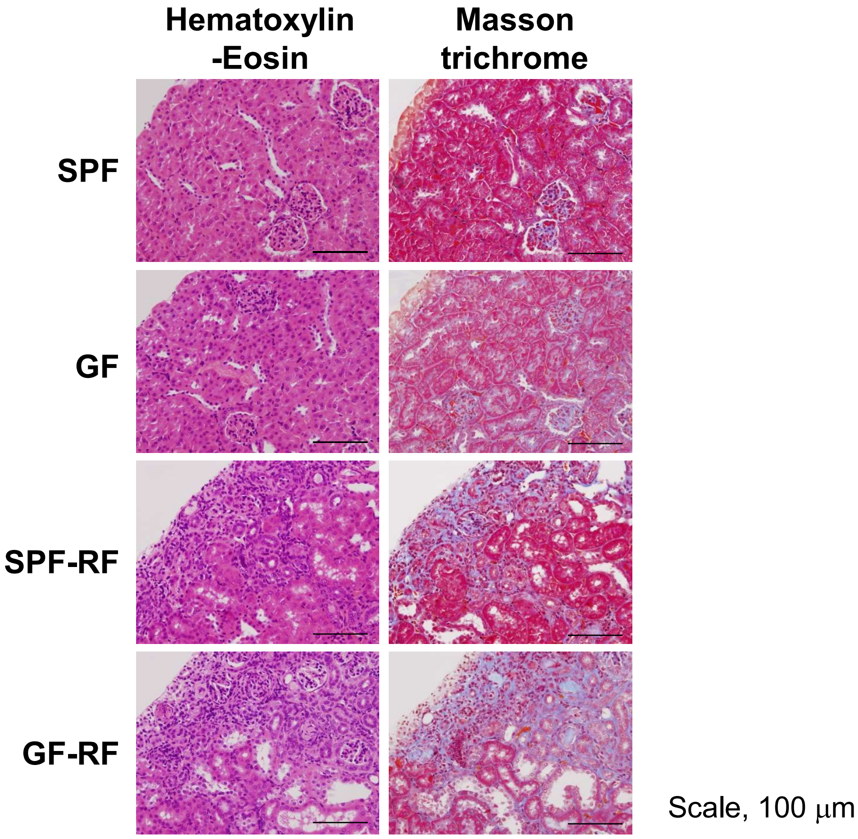

Supplementary Figure S5. Histologic images of kidney sections with Hematoxylin and eosin (H&E) and Masson's trichrome staining. Bar = 100 μm.

Notice; these results are reanalysis data conducted on the sample which were collected at previous work and stored at -80°C in our laboratory. (cf. *Kidney Int.*, 1–12 , 2017).

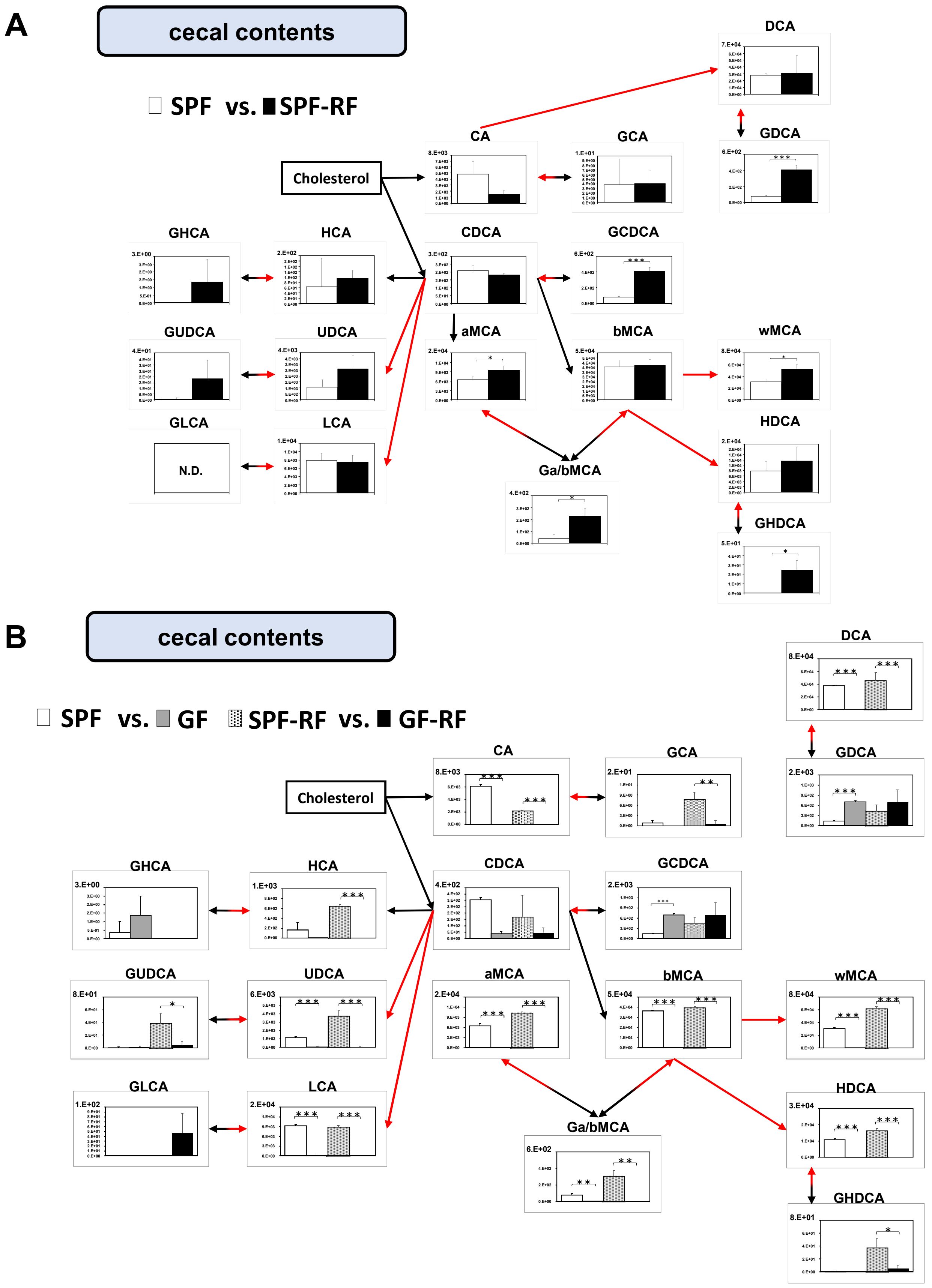

Supplementary Figure S6. Reconstruction of bile acid metabolic pathways from 18 bile acids
Relative quantitative analysis of the bile acids in cecal contents between (A) SPF and SPF-RF groups. (B) SPF and GF groups, SPF-RF and GF-RF groups.
Metabolic pathways are involved in host and microbiota as indicated by black arrows and red arrows, respectively. Bars indicate means ± SD of ion peak area in triplicate measurements. **P* < 0.05, ***P* < 0.01, ****P* < 0.001 compared between indicated groups (Student's t test). The pooled samples were prepared from each groups of mice; N = 4 in SPF control (SPF) and GF control (GF), and N = 3 in SPF renal failure (SPF-RF), and N = 5 GF renal failure (GF-RF) mice.

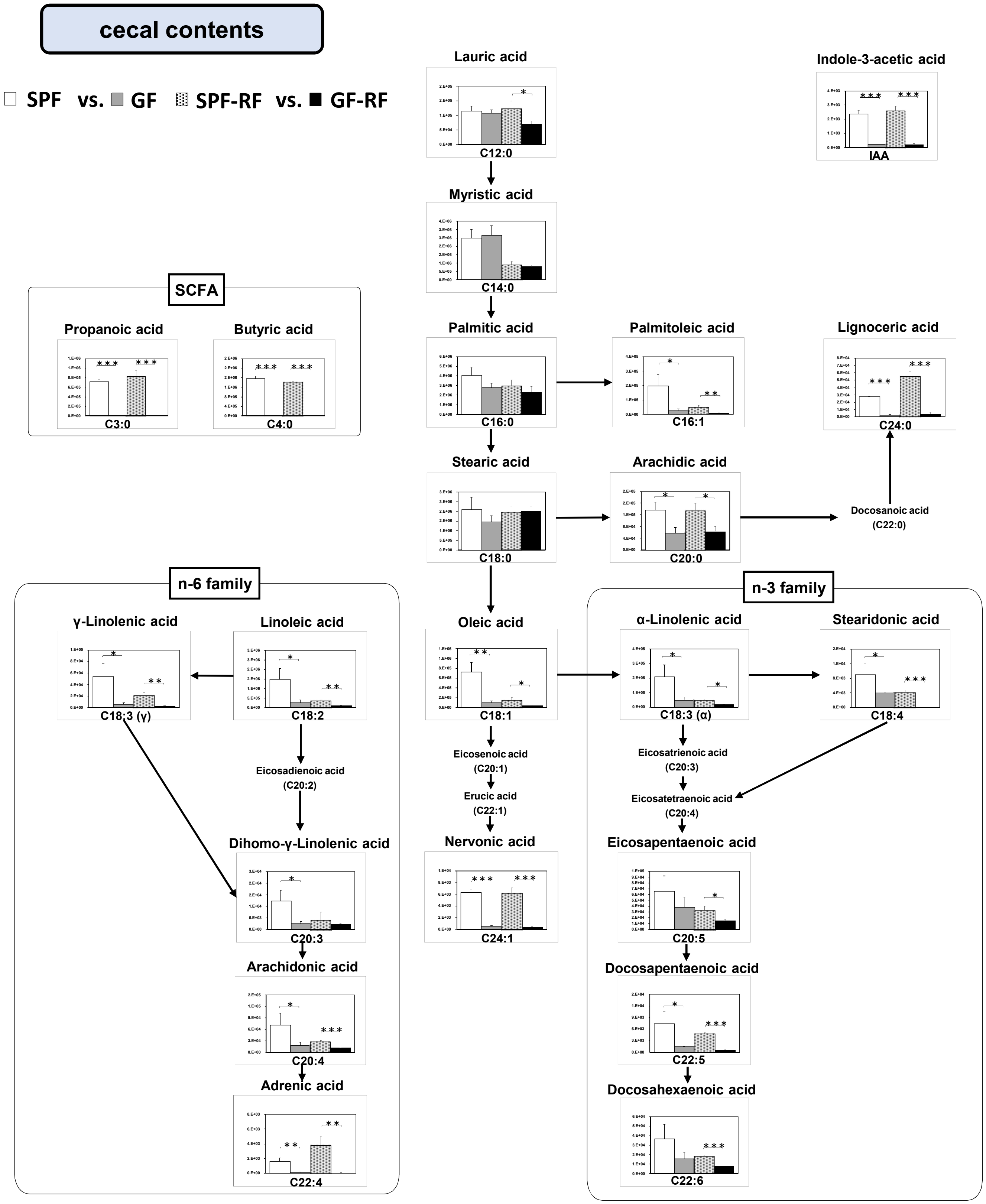

Supplementary Figure S7. Reconstruction of integrate metabolic pathways from 19 fatty acids, SCFA, and IAA

Relative quantitative analysis of the fatty acids and IAA in cecal contents SPF and GF groups, SPF-RF and GF-RF groups. Bars indicate means ± SD of ion peak area in triplicate measurements. *P < 0.05, **P < 0.01, ***P < 0.001 compared between indicated groups (Student's t test).

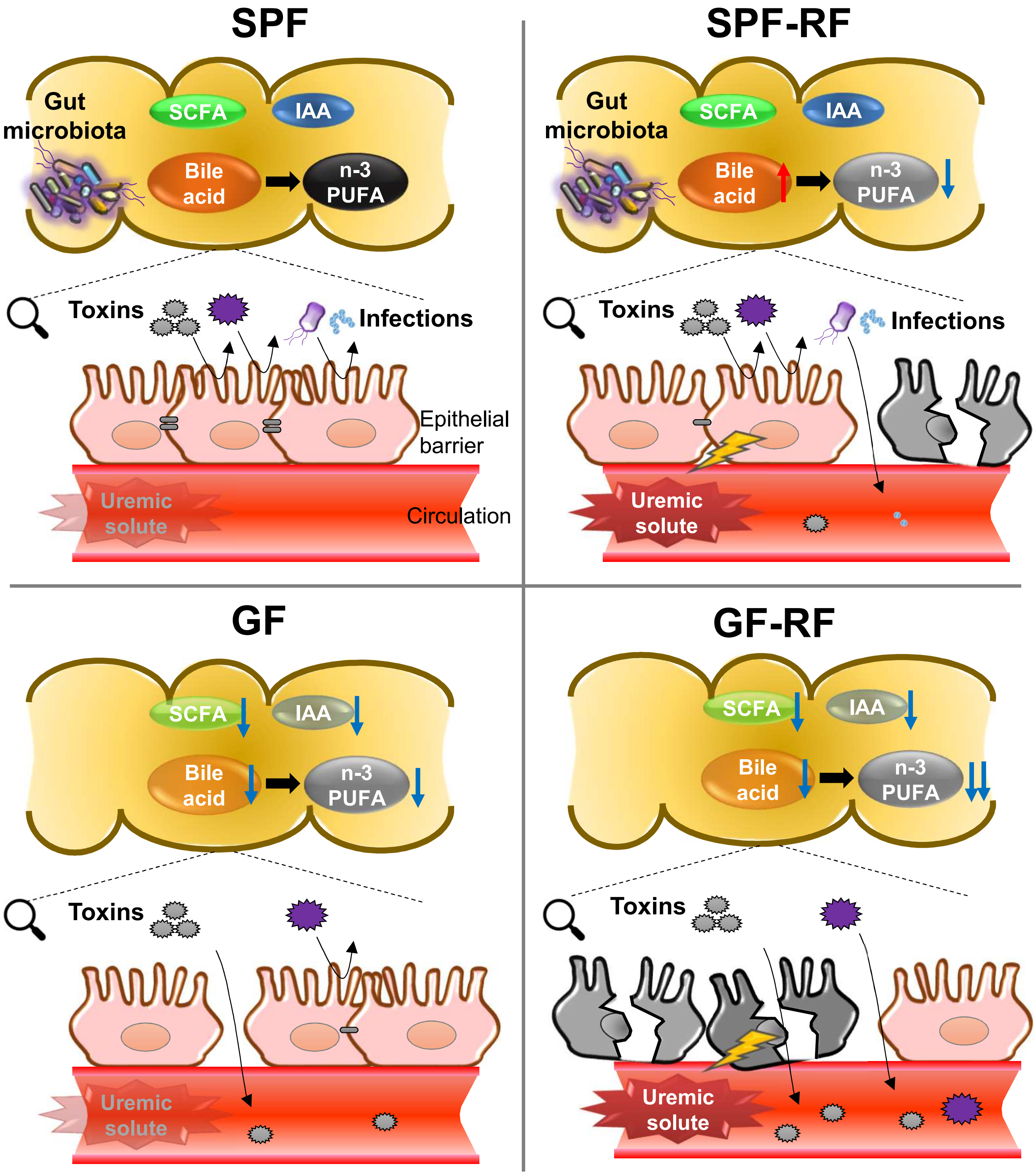

Supplementary Figure S8. A schematic model of the generation and protective effect of microbiota-derived carboxyl-containing metabolites in SPF, SPF-RF, GF, and GF-RF mice. Gut microbiota produces bile acids, SCFA, PUFA and IAA. Fatty acid is also derived from dietary sources. Germ-free conditions have deleterious effects on the intestinal carboxyl-containing metabolites level. Loss of the intestinal bile acids, n-3 PUFA, SCFA, and IAA accelerates epithelial barrier disruption, and induces inflammatory processes and the progression of renal failure in GF-RF mice.
